# Time to complete hepatitis C cascade of care among patients identified during mass screening campaigns in rural Rwanda: a retrospective cohort study

**DOI:** 10.1186/s12879-022-07271-z

**Published:** 2022-03-21

**Authors:** Innocent Kamali, Fabienne Shumbusho, Dale A. Barnhart, Françoise Nyirahabihirwe, Jean de la Paix Gakuru, Wellars Dusingizimana, Esdras Nizeyumuremyi, Placide Habinshuti, Stephen Walker, Jean Damascene Makuza, Janvier Serumondo, Gallican Nshogoza Rwibasira, Jean d’Amour Ndahimana

**Affiliations:** 1 Partners In Health / Inshuti Mu Buzima, Rwinkwavu, Rwanda; 2 Rwanda Military Hospital, Kigali, Rwanda; 3 Department of Global Health and Social Medicine, Harvard Medical School, Boston, MA USA; 4 Ministry of Health, Rwinkwavu District Hospital, Rwinkwavu, Rwanda; 5 GlaxoSmithKline, Philadelphia, PA USA; 6 STIs and OBBI Division, Rwanda Biomedical Centre, HIV/AIDS, Kigali, Rwanda; 7 School of Population and Public Health, University of British Columbia, Vancouver, BC Canada; 8 British Columbia Center for Disease Control, Vancouver, BC Canada

**Keywords:** Cascade of care, Hepatitis C, Rural health, Viral load, DAAs, Rwanda

## Abstract

**Background:**

Since the discovery of direct-acting antivirals, treatment for hepatitis C virus (HCV) is increasingly accessible in low-resource settings, but quality of care in these settings is not known. We described progression through the cascade of care among individuals who screened positive for HCV antibodies during a mass screening campaign in Kirehe and Kayonza, two rural Rwandan districts, in September 2019.

**Methods:**

This retrospective cohort study used routine clinical data to assess proportions of participants completing each stage of the cascade of care, including: (a) screening positive on rapid diagnostic test; (b) return of initial viral load results; (c) detectable viral load; (d) treatment assessment; (e) treatment initiation; (f) return of sustained virological response (SVR12) results; and (g) achieving SVR12. We proposed three indicators to assess timely care provision and used medians and interquartile ranges (IQR) to describe the time to complete the cascade of care.

**Results:**

Overall, 666 participants screened HCV positive, among them, 452 (68.1%) were female and median age was 61 years (IQR: 47, 70). Viral load results were returned for 537 (80.6%) participants of whom 448 (83.4%) had detectable viral loads. Of these, 398 (88.8%) were assessed for treatment, 394 (99%) were initiated, but only 222 (56.3%) had results returned for SVR12. Among those with SVR12 results, 208 (93.7%) achieved SVR12. When assessing timely care provision, we found 65.9% (95% CI: 62.0, 69.7) of initial viral load results were returned ≤ 30 days of screening; 45% (95% CI: 40.1, 49.8) of people with detectable viral load completed treatment assessment ≤ 90 days of initial viral load results; and 12.5% (95% CI: 9.2, 16.3) of SVR12 results were returned ≤ 210 days of treatment initiation among those who initiated treatment. The overall median time from screening to SVR12 assessment was 437 days.

**Conclusion:**

Despite high rates of SVR12 among those who completed all stages of the cascade of care, we identified gaps and delays in the treatment cascade. Improving communication between viral load testing hubs and health facilities could reduce the turn-around time for viral load testing, and actively monitor timeliness of care provision could improve quality of HCV care.

## Introduction

Globally, hepatitis C virus (HCV) affects 58 million people [1]. HCV increases the risk of developing liver cirrhosis and liver cancer and kills approximately 399,000 people annually [2]. According to recent estimates, 15% of the global burden of HCV infection is in Africa, where it affects more than 11 million people, 140,000 of whom live in Rwanda [3, 4]. Until the discovery of direct-acting antivirals (DAAs), treatment for HCV with interferon-based drugs was expensive, had poor efficacy, and often resulted in severe side-effects, making it practically impossible to treat the majority of people with HCV [4]. Through DAA treatment, over 95% of HCV patients may be able to achieve a Sustained Virologic Response (SVR12), also known as virologic cure, which occurs when there is no detectable HCV ribonucleic acid (RNA) twelve weeks after treatment completion [5]. DAAs have proved to be efficient and safe with high cure rates in the management of the disease in both high and low-income countries [6, 7].The recent reduction in the price of DAAs in low and middle income countries LMICs has made it possible to plan for a widespread treatment in those settings [8].

In Rwanda, the viral hepatitis program was established in 2011, and in 2018 the national hepatitis elimination plan announced an ambitious target to treat 90% of those infected by 2024 [9]. Subsequently, the Rwandan Ministry of Health introduced rapid diagnosis testing for HCV in all health facilities, capability to assess HCV RNA viral loads in 15 laboratory hubs, and free DAA treatment in all district hospitals and health centres. Since 2017, the Ministry of Health has organized national viral hepatitis mass screening campaigns for people aged 15 years and above as a strategy to identify patients who are HCV positive and link them to care. At the time of the launch of Rwanda’s hepatitis elimination plan, in December 2018, over 700,000 people had been screened and 9000 had been initiated to treatment, and the national program has since reported, a 92% treatment success rate (i.e. achieving SVR12) [10].

Despite the rapid scale-up of testing and existence of effective treatment for hepatitis C in Rwanda, achieving national elimination targets will require over 100,000 Rwandans to receive treatment for hepatitis C by 2024 [9]. To date, there is limited information about the time it takes for patients who screen positive for hepatitis C in mass screening campaigns to be enrolled, initiated to DAA treatment, and assessed for a cure. While the gap between the number of Rwandans receiving hepatitis screening and those receiving treatment suggests that delays in care provision are likely occurring, it is unclear which stages of the cascade are most susceptible to delays and bottlenecks. To be effective, HCV screening campaigns should be coupled with timely initiation treatment of those chronically infected. Long delays in treatment initiation may result in loss-to-follow-up, which could undermine the efforts of the elimination campaign. Similarly, delays in assessing the outcome of treatment may prevent further management for those in need. A previous modeling study concluded that the imperfect follow-up during the HCV cascade of care can reduce the real-world effectiveness of HCV therapy by as much as 75% [11]. In this study, we aim to understand the cascade of care for HCV among patients in rural Rwanda, with an emphasis on understanding the time from screening to SVR12 ascertainment among HCV patients identified through mass screening campaigns. By identifying gaps and delays in care provision, we hope to identify strategies for HCV management programs in our study area and elsewhere in Rwanda.

## Methods

### Study setting

Our study was conducted in two rural Rwandan districts, Kirehe and Southern Kayonza, supported by Partners in Health/Inshuti Mu Buzima (PIH/IMB). PIH/IMB is a non-governmental organization which has been supporting the Rwandan Ministry of Health in health systems strengthening since 2005. In September 2019, the national government implemented a mass screening campaign for hepatitis B and C. All Rwandans age 15 years or older were eligible for voluntary participation in the mass screening campaign, and, the population was sensitized to the screening campaign with messages through mass media communications and community health workers. PIH/IMB supported the screening and linkage to care activities in Kirehe and Southern Kayonza. SD Bioline RDTs manufactured by Abbott Diagnostics Korea Inc. were used to detect HCV antibodies [12]. In Rwanda, the standard of care for people who were screened positive during a mass campaign is same-day collection of venous blood samples, which are transported to a testing hub for viral load testing. If the viral load is detectable (≥ 15 IU/mL), the patients are assessed for eligibility for treatment initiation on DAAs, with pregnant and lactating women being instructed to delay treatment and complex cases, such as patients with decompensated cirrhosis, being referred to a higher level health facility, following the national guidelines [13]. Most of patients are prescribed a 12-week course of the first-line DAA regimen Sofosbuvir 400 mg + Daclatasvir 60mg/30 mg; however, patients with decompensated cirrhosis are prescribed a 24-week course of the treatment. Twelve weeks after the completion of DAA treatment, a second viral load test is performed to assess SVR12 and to identify whether patients are cured or will need further management. At the time of the September 2019 screening campaign, HCV infection management was not yet decentralized at the primary level-health centres. To facilitate patients’ access to care during the period of the screening and linkage to care activities described in this study, PIH/IMB organized a mobile clinic where clinicians and lab technicians from district hospitals met with patients at their nearest health facilities. This approach allowed patients to attend their local primary-level health centres for same-day clinical consultations, lab exams and treatment initiation, as has been described in detail elsewhere [14].

### Study design and population

This is a retrospective cohort study among participants aged 15 years and above who screened positive for HCV antibodies during the September 2019 mass hepatitis screening campaign in Kirehe and Kayonza districts. We included two district hospitals supported by PIH/IMB together with their 25 affiliated health centres, eight from Rwinkwavu District Hospital (in Kayonza District) and seventeen from Kirehe. We excluded those with negative or indeterminate HCV screening results or who were not screened at a PIH/IMB-supported site.

### Data source

Routine clinical data were collected during the mass screening campaign by trained clinicians and included self-reported demographic data and clinical characteristics. Additional data on viral load testing, linkage to care, treatment initiation, and treatment outcomes were extracted from HCV patient files. For programmatic purposes, all data were digitalized and entered into a dedicated Research Electronic Data Capture (REDCap) database to support clinical management [15].

Data was extracted from REDCap and de-identified for analysis. Data was extracted on March 24th, 2021, 18 months after the completion of the screening campaign.

### Data analysis

We analyzed the socio-demographic characteristics of participants who screened positive for HCV antibodies, including district of residence, gender, age, and marital status categorized as single, married or cohabitating and widow or divorced. In addition, we described Ubudehe category, which reflects the socio-economic status of households. There are four Ubudehe categories, where category one reflects the poorest and category four reflects the wealthiest [16]. We also reported health insurance status, which was categorized as, community based health insurance (CBHI), locally known as Mutuelle; other public and private health insurances; or uninsured. We also described patients’ self-reported comorbidities at the time of screening, including heart disease, HIV, hypertension, diabetes and chronic renal failure and self-reported risk factors for known for HCV infection, including history of traditional surgical practice, surgery, multiple sex partners, viral hepatitis in the family, blood transfusion, and unhygienic medical or household practices and genocide survivor status. Those who reported a previous diagnosis of hepatitis B, hepatitis C, liver disease, or having previously been screened for hepatitis were grouped into a single category reflecting prior history of hepatitis or liver disease. We described categorical data using frequencies and percentages and continuous data using medians and interquartile ranges (IQR). We also reported the numbers and percentages of participants completing each stage of the cascade of care for HCV infection with a binomial exact 95% confidence interval (CI) for each indicator. Stages in the cascade of care included: (a) screening positive on a rapid diagnostic test; (b) return of initial viral load results; (c) having a detectable viral load; (d) being assessed for treatment; (e) initiating treatment; (f) return of results for a post-treatment viral load test (SVR12 results); and (g) achieving SVR12. To assess whether socioeconomic status was associated with progressing through the cascade of care, we used standard two-by-two tables to calculate the unadjusted risk ratio and 95% confidence intervals comparing the probability of progressing through each stage by ubudehe status dichotomized into low ubudehe (category one or two) and high ubudehe (category three) [17].

We defined three key indicators to assess timely provision of HCV care. These indicators included: (a) percentage of people who screened RDT positive for HCV who had their initial viral load test results returned within 30 days of screening; (b) percentage of people with detectable HCV RNA who completed their treatment assessment within 90 days of receiving their viral load results; and (c) the percentage of patients who initiated treatment who had their SVR12 results returned within 210 days of treatment initiation. For each indicator, patients who had not completed the stage of interest at the time of data analysis were included in the denominator, but not the numerator. Patients who were documented as having completed a stage but were missing data on dates such that it was not possible to classify them as having completed the stage of interest within the desired window were excluded from the complete case analysis. However, we considered two sensitivity analyses: a best-case scenario where all those who were missing relevant dates were considered to have experienced the event of interest within the desired time window and a worst-case scenario where all those who were missing relevant dates were considered to have experienced the event of interest after the desired time window. For each indicator, we reported proportions with their binomial exact 95% confidence interval (CI). Finally, we calculated median and IQRs to estimate the days required to complete key stages of the HCV infection cascade of care, including: (a) days between screening and return of initial viral load results; (b) days between return of initial viral load results and treatment initiation; (c) days between treatment initiation and return of SVR12 results and; (d) the overall days from screening to return of SVR12 results. People who were eligible to have completed a stage but had not yet completed it at the time of data analysis were considered to be right censored at the extreme end of the distribution and were included in the calculation of the median and IQR. All analyses were conducted using Stata v.15.1 (Stata Corp, College Station, TX, USA).

## Results

Of the 9240 people included in the REDCap database at the time of data extraction, 8776 (94.5%) were from PIH/IMB supported sites, and 1725 (19.6%) had been screened during the September 2019 campaign. Most of the remaining patients in the database reflect individuals who were screened during later screening campaign conducted by PIH/IMB after September 2019 campaigns and targeting specific populations, such as patients with non-communicable diseases’ (NCD). Of the 1725, we excluded 1057 (61.2%) people with HCV-RDT negative results and 2 people without HCV-RDT results for a final sample size of 666 participants. Because district officials often refer only RDT + cases to PIH/IMB, the proportion of HCV-RDT individuals in our database, does not reflect the prevalence of HCV in these districts. The majority of patients were from Kirehe district (n = 459, 68.9%) and most were female (n = 452, 68.1%) (Table 1). The median age was 61 years (IQR: 47,70). More than half of study participants were married or cohabitating (n = 357, 54.2%). Ubudehe category 3 was the most common (n = 310, 48.6%), and nearly all study participants used Mutuelle as their health insurance (n = 641, 96.4%). The most common self-reported comorbidities were heart disease (n = 33, 5.0%) and HIV (n = 20, 3.0%) respectively. The most common self-reported risk factor was history of traditional surgical practice (n = 215, 32.3%).

**Table 1:** Socio-demographic and clinical characteristics (N = 666)

Variable	N	%
District (N = 666)		
Kayonza	207	31.1
Kirehe	459	68.9
Sex (N = 664)		
Female	452	68.1
Male	212	31.9
Age, years (median (IQR), N = 661)	61 (47,70)	
Marital status (N = 659)		
Single	74	11.2
Married or cohabitating	357	54.2
Widowed/divorced	228	34.6
Ubudehe category (N = 638)		
Category 1	104	16.3
Category 2	224	35.1
Category 3	310	48.6
Insurance status (N = 665)		
Mutuelle	641	96.4
RSSB/RAMA, MMI, Others	22	3.3
No insurance	2	0.3
Co-morbidities (N = 666)^a^		
Heart disease	33	5.0
HIV	20	3.0
Hypertension	14	2.1
Diabetes	12	1.8
Chronic renal failure	11	1.7
HCV Risk factors (N = 666)^b^		
Traditional operation	215	32.3
Ever had surgery	55	8.3
Ever experienced physical trauma	51	7.7
Multiple sexual partners	44	6.6
Viral Hepatitis in the family	46	6.9
Blood transfusion or needle stick	32	4.8
Unhygienic medical or household practices	30	4.5
History of hepatitis diagnosis, liver disease, or screening (N = 666)	9	1.4

^a^Patients could report more than one comorbidity

^b^Patients could report more than one risk factor

**Fig. 1:**
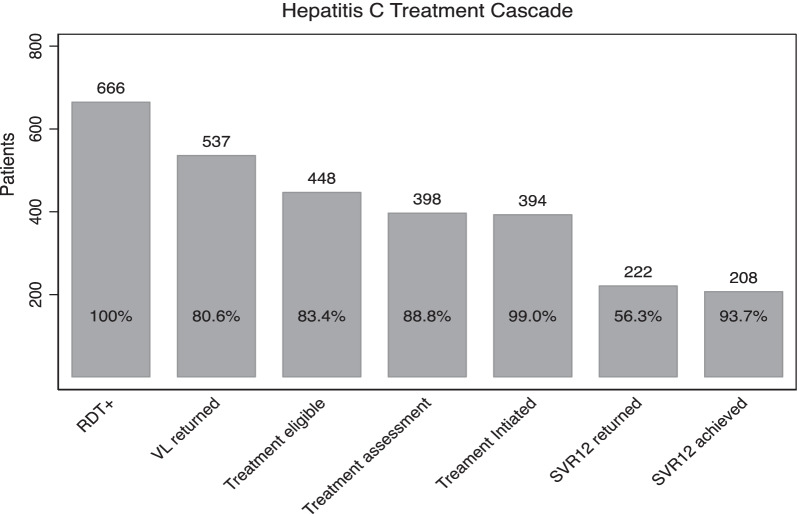
Cascade of care for management of hepatitis C among patients identified in the mass screening campaign. *RDT + : Rapid Diagnostic Test positive *VL: Viral load *SVR12: Sustained Virologic Response after treatment completion

Of the 666 people who screened HCV RDT positive, initial viral load results were returned for 537 (80.6%, 95% CI: 77.4–83.6) people (Fig. 1) Of those, 448 (83.4%, 95% CI: 80.0–86.5) had a detectable viral load and 398 (88.8%, 95% CI: 85.6–91.6) of patients with detectable viral loads were assessed for treatment. Almost all patients who were assessed for treatment, were initiated on treatment (n = 394, 99.0%, 95% CI: 97.4–99.7), but only 222 (56.3%, 95% CI: 51.3, 61.3) of patients who initiated treatment had results returned for their post-treatment SVR12 test. Among those 222 patients assessed, 208 (93.7%, 95% CI: 89.6, 96.5) achieved SVR12. We did not observe any associations between ubudehe status and progression through the cascade of care (Table 2).

**Table 2:** Progress through the hepatitis C treatment cascade by ubudehe category (socioeconomic status)

Cascade of care	Ubudehe One or Two Low socioeconomic status		Ubudehe Three High socioeconomic status		Risk ratio (95% CI)
	n/N	%	n/N	%	
RDT +	328/328	100	310/310	100	–^a^
VL returned	270/328	82.3	240/310	77.4	1.06 (0.98, 1.15)
Treatment eligible	228/270	84.4	193/240	80.4	1.05 (0.97, 1.14)
Treatment assessment	199/228	87.3	173/193	89.6	0.97 (0.91, 1.04)
Treatment initiated	197/199	99.0	171/173	98.8	1.00 (0.98, 1.02)
SVR12 returned	109/197	55.3	101/171	59.1	0.94 (0.78, 1.12)
SVR12 achieved	105/109	96.3	91/101	90.1	1.07 (0.99, 1.15)

*RDT + : rapid diagnostic test positive

*VL: vral load

*SVR12: sustained virologic response after treatment completion

^a^Study population was restricted to individuals who were RDT +

When assessing the key indicators for timely provision of HCV care, we found that 65.9% (95% CI: 62.0, 69.7) of people who screened positive had their initial HCV viral load results returned within 30 days of screening (Table 3). Among patients with a detectable viral load, 45.0% (95% CI: 40.1, 49.8) were assessed for treatment initiation within 90 days of receipt of viral load results. Only 12.5% (95% CI: 9.2, 16.3) of the participants who initiated treatment had their SVR12 results returned within 210 days of treatment initiation. Our findings were not sensitive to assumptions about the timing of events among individuals who were missing data on dates (Table 4). Overall, among individuals who initiated treatment, the median number of days from screening to return of SVR12 results was 437 days The median time between screening and return of initial viral load results was 15 days the median time between return of initial viral load results and treatment assessment was over three months (104 days); and the median time between treatment initiation and return of SVR12 results was almost 12 months (334 days).

**Table 3:** Proportion of patients receiving timely provision of care for hepatitis C

Indicator	N	%	95% CI
Proportion of initial HCV viral load results returned ≤ 30 days of screening among people who screened RDT +			
Complete case analysis^a^ (N = 594)	392	65.9	(62.0, 69.8)
Worst case scenario^b^ (N = 666)	392	58.9	(55.0, 62.6)
Best case scenario^c^ (N = 666)	464	69.7	(66.0, 73.1)
Proportion of patients who were assessed for treatment eligibility ≤ 90 days after receiving a viral load results among those with a detectable viral load			
Complete case analysis^a^ (N = 420)	189	45.0	(40.2, 49.9)
Worst case scenario^b^ (N = 448)	189	42.2	(37.7, 46.9)
Best case scenario^c^ (N = 448)	217	48.4	(43.7, 53.2)
Proportion of SVR12 results returned ≤ 210 days of treatment initiation among patients who initiated treatment			
Complete case analysis^a^ (N = 368)	46	12.5	(9.3, 16.3)
Worst case scenario^b^ (N = 394)	46	11.7	(8.7, 15.23
Best case scenario^c^ (N = 394)	72	18.3	(14.6, 22.5)

^a^Complete case analyses excluded individuals who completed the stage of interest, but were missing data on the relevant start or end dates necessary to assess timing of the event

^b^The worst-case scenario assumed that all individuals who were missing relevant dates had experienced the event of interest after the cut off

^c^The best-case scenario assumed that all individuals missing relevant dates experienced the event of interest before the cut-off

**Table 4:** Median time to complete key stage of the hepatitis C cascade of care

Variable	N	Median^a^	(IQR)^a^	
Time between screening and return of initial viral load results	594	15	13	61
Time between return of initial viral load results and assessment for treatment eligibility	420	104	66	123
Time between treatment initiation and return of SVR12 results	368	334	287	–^b^
Time between screening and SVR12 and return of SVR12 results among those who initiated treatment	368	437	406	–^b^

All durations are defined in days

^a^The lower bound of the IQR, median, and upper bound of the IQR can be conceptualized as the points at which at least 25%, 50%, and 75% of the population had completed each stage of the cascade of care, respectively. ^b^Less than 75% of the eligible population reached this milestone; so, the 75th percentile is not defined

## Discussion

In accordance with the WHO goal of eliminating HCV as a public health threat by 2030, countries worldwide are expanding access to HCV diagnosis and treatment services [8]. However, to ensure HCV elimination, these programs must complete the full cascade of care for HCV in a timely fashion. Our patients were older and more likely to be female than the general population. As has been reported elsewhere, age is a strong risk factor for hepatitis C in Rwanda [18]. The association between hepatitis C and age also explains the large proportion of female patients, since there is pronounced gender imbalance with more female than males among older age groups in Rwanda [19]. Our patients were also more likely to be insured than the general population (83%) [19]. However, our patients were similar to the general population in terms of ubudehe status (16% of the general population is in ubudehe 1, 36% in ubudehe 2, and 45% in ubudehe 3) [20] and HIV co-infection (3.0% in the general population) [21]. Among the 666 patients who were identified as anti-HCV positive during the September 2019 screening campaign, only 222 had their SVR12 results returned by March 2021. The two biggest gaps in the cascade of care were return of initial viral load results and the return of SVR12 results. During the September 2019 screening campaign, viral load samples were collected on the same day as RDT administration. Consequently, we believe that missing or delayed initial viral load results could be explained by poor communication between the health facilities and the testing hub. The proportion of patients whose SVR12 test results were returned was even lower (56.3%), which likely reflects both poor communication between health facilities and viral load hubs and challenges in following up with patients for sample collection. This aligns with the findings from Nsanzimana et al., where 34% of patients who completed HCV treatment were found to either be lost to follow-up for SVR12 assessment or to not having HCV RNA results returned [7]. These gaps point to a need to improve the overall laboratory communication and turnaround time, which has previously been reported to be an important element impacting provider performance and patient care [22, 23]. Critically, we did not observe that patients from the lower ubudehe categories, reflecting a lower socioeconomic status, were less likely to progress through the treatment cascade. As previously noted, the mobile clinic approach used to link our patients to care has been estimated to reduce patients’ costs to accessing treatment by almost 10 USD, which is a meaningful reduction in rural Rwanda and may have removed economic barriers to accessing care for some patients [14].

Although viral load testing was a barrier to provision of care, our treatment initiation and treatment assessment rates were quite high. These outcomes were achieved in the context of a mobile clinical campaign, which has previously been reported as a low-cost strategy to support linkage to care, and may be a scalable model for improving patients’ access to care [14]. Similarly, among patients whose SVR12 results were returned, we found that HCV cure rate was very high (93.5%). This level slightly exceeds levels previously reported in Rwanda among treatment naïve patients with non-cirrhotic or with compensated cirrhosis treated with Ledipasvir 90 mg/sofosbuvir 40 mg (87%) [24] and among a combination of treatment naïve and experienced patients treated through the national program (92%) [7]. Our results reinforce previous findings that treatment for HCV can be successfully implemented in low-income countries and among individuals living in limited resources settings [25]. However, this high rate of treatment success does not diminish the importance ensuring that all patients complete the full cascade of care. HCV genotyping is not part of Rwanda’s current standard of care for HCV treatment, so we do not know the HCV genotypes for these patients. However, in a previous open trial of DAAs in Rwanda, 16% of participants had genotype 4r, which has previously been associated with resistance to DAA regimens consisting of ledipasvir or sofosbuvir [24].The high prevalence of this resistant genotype in our setting underscores the importance of ensuring that all HCV patients complete treatment, ascertain their SVR12 status, and are referred to second line treatment, as needed.

Among patients who completed treatment, it took over 14 months to complete the full HCV cascade of care. In general, the time to complete individual stages of the cascade of care compares favorably to what has been reported in other similar settings. For example, while in our study the median time to receive initial viral load results was 15 days, in Malawi’s HIV program, the median turn-around time is almost 3months [26]. Our median time between return of initial viral test results and treatment assessment (104 days) is very similar to what has been reported in an HCV program at a tertiary hospital in the USA (107 days) [27] and better than what was found in another HCV program at a tertiary care centre in the USA (300 days) [28]. However, these studies were conducted in routine care settings, while our data reflects treatment initiation following a mass screening campaign and targeted mobile clinic outreach program to support decentralized care. In Rwandan settings where HCV infected patients do not have access to this targeted, decentralized outreach program as the one offered to the patients in this study by the PIH/IMB program, gaps in the cascade of care are likely even larger and delays in service provision are likely even longer than reported here. Addressing these delays will be critical for Rwanda to achieve its national target of treating 90% of those infected with HCV by 2024. Although Rwandan guidelines allowed decentralized management of hepatitis B and C and its integration into routine care at the health centre level in August 2020 [29]; in practice, many health centre nurses may not have the necessary experience to provide these services and many health centers may not have the reagents and machines needed to conduct pre-treatment testing and identify complex cases. Achieving successful decentralization will require sustained investment in nurse training and mentorship, investment in and financing for laboratory testing at the health center level, and investment in the monitoring and evaluation of patient outcomes.

We proposed three indicators for assessing timely provision of hepatitis C care: (a) proportion of initial HCV viral load results returned ≤ 30 days of screening among people who screened RDT + ; (b) proportion of patients who were assessed for treatment eligibility ≤ 90 days after receiving a viral load results among those with a detectable viral load; and (c) proportion of SVR12 results returned ≤ 210 days of treatment initiation among patients who initiated treatment. In a health system where each of these indicators are met, a patient could expect to complete treatment for HCV in less than 11 months from screening. A second strategy that could reduce turn-around time for lab results and eliminate the risk of poor communication between testing hubs and health facilities is the introduction of point of care tests to assess hepatitis C viraemia. There are currently two major approaches to providing point of care testing. The first replaces viral RNA test with core antigen tests, which can provide results in 60 min [30–32]. Core antigen tests have been approved by the WHO as an alternative for confirmatory viral load testing [33, 34], and may also provide an alternative approach for assessing SVR12 [35].

The second uses GeneXpert to provide point-of-care viral load testing. Similar applications of GeneXpert have achieved success in HIV programs in limited-resource settings, as they allow patients to receive viral load results on the same day as sample collection [25], and have been successfully used in HCV pilot programs in Egypt [36], Indonesia [37], and Tanzania [38]. Given that HCV GeneXpert is WHO pre-qualified for HCV viral load testing, it provides an opportunity for improving HCV patients monitoring and would also allow for integrated multi-disease testing platforms [39].

Our study has some limitations. First, our results are not fully generalizable as they focused on catchment areas for two district hospitals that receive substantial support from PIH/IMB. As noted, these catchment areas were serviced by a targeted mobile clinic to support HCV treatment initiation during the study period. As discussed above, we anticipate that this limitation means that the observed gaps or delays in care provision in our setting would be lower than elsewhere in Rwanda. Second, the cascade of care for the patients in our study was interrupted by the COVID-19 pandemic. However, most patients were initiated through the mobile clinic program, which was active between November 2019 and January 2020 and preceded the COVID-19-related national lockdown in Rwanda, which occurred between 14th March 2020 and 3rd May 2020. Furthermore, SVR12 follow-up for most patients should have occurred between January and March 2021, during which time no lockdowns or restrictions on intra-district travel were in place and patients should have had uninterrupted access to their local health facilities. Third, our study used routine clinical records, which are subject to missing data, particularly on dates for steps of cascade of care. However, we assessed the sensitivity of our results to missing data and did not find that it meaningfully changed our results. Similarly, because patients do not routinely attend the health facility between the time of receiving their last prescription for DAAs and the time of their SVR12 testing, we cannot estimate the proportion of patients who did not complete treatment or the proportion who completed treatment but did not return for SVR12 testing. Fourth, adherence assessment was not conducted as part of this retrospective analysis, which relied on the use of existing routine clinical data. However, the study team is currently conducting a prospective cohort to validate DAA adherence tools and assess the association between adherence and treatment success. Fifth, our recommended quality of care indicators, “Proportion of SVR12 results returned ≤ 210 days of treatment initiation among patients who initiated treatment” does not account for the fact that some patients could have been prescribed a 24 week course of treatment and would not be eligible to receive SVR12 testing until at least 270 days after treatment initiation. Treatment duration was not readily available from our clinical database, however, based on previous estimates of higher APRI (Aspartate Aminotransferase to Platelet Ratio Index) score among people initiating HCV treatment elsewhere in Rwanda [16], we believe that fewer than ten percent of patients were prescribed a 24 week-course treatment. In the interest of proposing a single indicator that would be easy to implement for the monitoring of future programs, we do not recommend disaggregating this indicator by treatment duration.

## Conclusion

Although HCV treatment was successful in rural Rwanda, we identified some gaps and delays among patients’ progress through the cascade of care. The major gaps and delays were related to viral load testing, suggesting that shortening the turnaround time for viral load results and improving communication between testing hubs and health facilities could improve patient outcomes and strengthen the national HCV elimination program. Our proposed indicators for timely provision of HCV care could be used by future screening programs to monitor their quality of care.

## Data Availability

The datasets used and/or analyzed during the current study are available from the corresponding author on reasonable request.

## Abbreviations

CBHI: Community Based Health insurance
CI: Confidence Interval
DAAs: Direct Acting antivirals
HCV: Hepatitis C virus
IHDPC: Institute of HIV disease prevention and control
IMBRC: Inshuti Mu Buzima Research Committee
IQR: Interquartile Range
PIH/IMB: Partners In Health/Inshuti Mu Buzima
LMIC: Low and Middle Income Countries
MoH: Ministry of Health
NCD: Non Communicable Diseases
SVR12: Sustained Virologic Response after 12 weeks
RNEC: Rwanda National Ethics Committee
RDT: Rapid Diagnostic Test
RBC: Rwanda Biomedical Centre
REDCap: Research Electronic Data Capture
RNA: Ribonucleic Acid
RNEC: Rwanda National Ethics Committee
WHO: World Health Organization

## References

[1] World Health Organization. Global progress report on HIV, viral hepatitis and sexually transmitted infections, 2021. Accountability for the global health sector strategies 2016–2021: actions for impact. Geneva, Switzerland; 2021. Report No.: ISBN 978-92-4-002707-7. https://www.who.int/publications/i/item/9789240027077.

[2] Blach S, Zeuzem S, Manns M, Altraif I, Duberg A-S, Muljono DH (2017). Global prevalence and genotype distribution of hepatitis C virus infection in 2015: a modelling study. Lancet Gastroenterol Hepatol.

[3] Ministry of Health, Rwanda Biomedical Centre. Annual report for HIV and Viral Hepatitis. Kigali, Rwanda; 2018. https://rbc.gov.rw/fileadmin/user_upload/report2019/report2019/Annual%20Report%20for%20HIV%20and%20Viral%20Hepatitis%202017-2018.pdf.

[4] Welsch C, Jesudian A, Zeuzem S, Jacobson I (2012). New direct-acting antiviral agents for the treatment of hepatitis C virus infection and perspectives. Gut.

[5] Vermehren J, Park JS, Jacobson IM, Zeuzem S (2018). Challenges and perspectives of direct antivirals for the treatment of hepatitis C virus infection. J Hepatol.

[6] Roche B, Coilly A, Duclos-Vallee JC, Samuel D (2018). The impact of treatment of hepatitis C with DAAs on the occurrence of HCC. Liver Int.

[7] Nsanzimana S, Penkunas MJ, Liu CY, Sebuhoro D, Ngwije A, Remera E (2020). Effectiveness of direct-acting antivirals for the treatment of chronic hepatitis C in Rwanda: a retrospective study. Clin Infect Dis.

[8] World Health Organization (2018). Progress report on accessto hepatitis c treatment: focus on overcoming barriers in low-and middle-income countries.

[9] Umutesi G, Shumbusho F, Kateera F, Serumondo J, Kabahizi J, Musabeyezu E (2019). Rwanda launches a 5-year national hepatitis C elimination plan: a landmark in sub-Saharan Africa. J Hepatol.

[10] Ministry of Health. Rwanda takes the lead in Sub-Saharan Africa to eliminate Hepatitis C. 2018; https://www.moh.gov.rw/news-detail/rwanda-takes-the-lead-in-sub-saharan-africa-to-eliminate-hepatitis-c.

[11] Linas BP, Barter DM, Leff JA, Assoumou SA, Salomon JA, Weinstein MC (2014). The hepatitis C cascade of care: identifying priorities to improve clinical outcomes. PLoS ONE.

[12] Jargalsaikhan G, Eichner M, Boldbaatar D, Bat-Ulzii P, Lkhagva-Ochir O, Oidovsambuu O (2020). Sensitivity and specificity of commercially available rapid diagnostic tests for viral hepatitis B and C screening in serum samples. PLoS ONE.

[13] Ministry of Health, Rwanda Biomedical Centre. National guidelines for prevention and management of viral hepatitis B, C and sexually transmitted infections. 2019.

[14] Kamali I, Barnhart DA, Nyirahabihirwe F, de la Paix GJ, Uwase M, Nizeyumuremyi E (2021). Initiation of hepatitis C treatment in two rural Rwandan districts: a mobile clinic approach. BMC Infect Dis.

[15] Harris PA, Taylor R, Thielke R, Payne J, Gonzalez N, Conde JG (2009). Research electronic data capture (REDCap)—a metadata-driven methodology and workflow process for providing translational research informatics support. J Biomed Inform.

[16] Chika Ezeanya-Esiobu. The rise of homegrown ideas and grassroots voices. New directions in social policy in Rwanda. Geneva, Switzerland; 2017 May. Report No.: 2017–6. https://www.unrisd.org/unrisd/website/document.nsf/(httpPublications)/3AC45BEF8587AD6AC1258122003E9475?OpenDocument.

[17] Morris JA, Gardner MJ (1988). Statistics in medicine: calculating confidence intervals for relative risks (odds ratios) and standardised ratios and rates. BMJ.

[18] Makuza JD, Liu CY, Ntihabose CK, Dushimiyimana D, Umuraza S, Nisingizwe MP (2019). Risk factors for viral hepatitis C infection in Rwanda: results from a nationwide screening program. BMC Infect Dis.

[19] National Institute of statistics. Rwanda Demographic and Health Survey 2019–20. Kigali, Rwanda; 2021. https://www.statistics.gov.rw/datasource/demographic-and-health-survey-dhs.

[20] Rwanda National Institute of Statistics. Comprehensive food security , vulnerability and nutrition analysis. Kigali, Rwanda; 2018. https://www.statistics.gov.rw/datasource/comprehensive-food-security-and-vulnerability-and-nutrition-analysis-survey-cfsva.

[21] Rwanda Biomedical Centre (RBC). Rwanda population-based HIV Impact Assessment (RPHIA) 2018–2019: Final Report. Kigali: RBC; 2020 Sep. http://phia.icap.columbia.edu.

[22] Holland LL, Smith LL, Blick KE (2005). Reducing laboratory turnaround time outliers can reduce emergency department patient length of stay: an 11-hospital study. Am J Clin Pathol.

[23] Dey B, Bharti JN, Chakraborty M (2013). Laboratory turnaround time. Int J Health Sci.

[24] Gupta N, Mbituyumuremyi A, Kabahizi J, Ntaganda F, Muvunyi CM, Shumbusho F (2019). Treatment of chronic hepatitis C virus infection in Rwanda with ledipasvir–sofosbuvir (SHARED): a single-arm trial. Lancet Gastroenterol Hepatol.

[25] Abozeid M, Alsebaey A, Abdelsameea E, Othman W, Elhelbawy M, Rgab A (2018). High efficacy of generic and brand direct acting antivirals in treatment of chronic hepatitis C. Int J Infect Dis.

[26] Minchella PA, Chipungu G, Kim AA, Sarr A, Ali H, Mwenda R (2017). Specimen origin, type and testing laboratory are linked to longer turnaround times for HIV viral load testing in Malawi. PLoS ONE.

[27] Tabba OM, Tenzi C, Mohamed AM, James LH. Timeline of linkage to care in hepatitis C: from screening to treatment in the outpatient setting at tertiary care centre. 2018.

[28] Kwo PY, Puenpatom A, Zhang Z, Hui SL, Kelley AA, Muschi D (2019). Initial uptake, time to treatment, and real-world effectiveness of all-oral direct-acting antivirals for hepatitis C virus infection in the United States: a retrospective cohort analysis. PLoS ONE.

[29] Rwanda Biomedical Centre. Addendum to the national guidelines for prevention and management of viral hepatitis B, C and Sexually transmitted infections. 2020.

[30] Rockstroh JK, Feld JJ, Chevaliez S, Cheng K, Wedemeyer H, Sarrazin C (2017). HCV core antigen as an alternate test to HCV RNA for assessment of virologic responses to all-oral, interferon-free treatment in HCV genotype 1 infected patients. J Virol Methods.

[31] Pérez-García A, Aguinaga A, Navascués A, Castilla J, Ezpeleta C (2019). Hepatitis C core antigen: diagnosis and monitoring of patients infected with hepatitis C virus. Int J Infect Dis.

[32] Wong XZ, Gan CC, Mohamed R, Yahya R, Ganapathy S, Tan SS (2020). Hepatitis C core antigen testing to diagnose active hepatitis C infection among haemodialysis patients. BMC Nephrol.

[33] World Health Organization (2017). Guidelines on hepatitis B and C testing.

[34] Wang J-H, Chen C-H, Chang C-M, Feng W-C, Lee C-Y, Lu S-N (2020). Hepatitis C virus core antigen is cost-effective in community-based screening of active hepatitis C infection in Taiwan. J Formos Med Assoc.

[35] Lin SF, Tung S-Y, Wei K-L, Chen C-H, Hu T-H, Shen CH (2020). Clinical utility of hepatitis C virus core antigen assay in the monitoring of direct-acting antivirals for chronic hepatitis C. PLoS ONE.

[36] Shiha G, Soliman R, Serwah A, Mikhail N, Asselah T, Easterbrook P (2020). A same day ‘test and treat’ model for chronic HCV and HBV infection: results from two community-based pilot studies in Egypt. J Viral Hepat.

[37] Thedja MD, Wibowo DP, El-Khobar KE, Ie SI, Turyadi, Setiawan L, et al. Improving linkage to care of hepatitis C: clinical validation of GeneXpert® HCV viral load point-of-care assay in Indonesia. Am J Trop Med Hyg. 2021. https://www.ajtmh.org/view/journals/tpmd/aop/article-10.4269-ajtmh.20-1588/article-10.4269-ajtmh.20-1588.xml. Accessed 10 Jun 2021. 10.4269/ajtmh.20-1588 PMC8274760 33999849

[38] Mohamed Z, Mbwambo J, Rwegasha J, Mgina N, Doulla B, Mwakale P (2020). In-field evaluation of Xpert® HCV viral load Fingerstick assay in people who inject drugs in Tanzania. Liver Int.

[39] Peeling RW, Boeras DI, Marinucci F, Easterbrook P (2017). The future of viral hepatitis testing: innovations in testing technologies and approaches. BMC Infect Dis.

